# European multicenter study on LOH of APOC3 at 11q23 in 766 breast cancer patients: relation to clinical variables

**DOI:** 10.1038/sj.bjc.6690435

**Published:** 1999-05

**Authors:** V Launonen, K Laake, P Huusko, D Niederacher, M W Beckmann, R B Barkardottir, E K Geirsdottir, J Gudmundsson, P Rio, Y-J Bignon, S Seitz, S Scherneck, I Bièche, M-H Champème, D Birnbaum, G White, J Varley, M Sztán, E Olah, A Osorio, J Benitez, N Spurr, N Velikonja, B Peterlin, Å Borg, A-M Cleton-Jansen, P Devilee, R Bloigu, R Lidereau, A-L Børresen-Dale, R Winqvist, the Breast Cancer Somatic Genetics Consortium

**Affiliations:** Departments of Clinical Genetics, Mathematical Science/Statistics, Oncology and Radiotherapy, University of Oulu/Oulu University Hospital, Oulu, Finland; Department of Genetics, Institute for Cancer Research, The Norwegian Radium Hospital, Oslo, Norway; Department of Obstetrics and Gynecology, Heinrich-Heine-University, Düsseldorf, Germany; Laboratory of Cell Biology, Department of Pathology, University Hospital of Iceland, Reykjavik, Iceland; INSERM U 484 Clermont-Ferrand, France; Laboratoire d'Oncologie Moléculaire INSERM CRI 9502 and EA 2145, Centre Jean Perrin, Clermont-Ferrand, France; Department of Tumour Genetics, Max-Delbrück-Centrum, Berlin, Germany; Centre René Huguenin, Saint-Clod, France; Institut Paoli-Calmettes and U.119 INSERM, Marseille;, France; Section of Molecular Genetics, Paterson Institute for Cancer Research, Manchester, UK; National Institute of Oncology, Budapest, Hungary; Department of Genetics, Fundación Jiménez Díaz, Madrid, Spain; Human Genetic Resources, ICRF Laboratories, London, UK; Division of Medical Genetics, Department of Obstetrics and Gynaecology, Ljubjana, Slovenia; Institute of Oncology, University Hospital, Lund, Sweden; Department of Pathology, Leiden University, Leiden, The Netherlands

**Keywords:** breast cancer, chromosome 11q23, loss of heterozygosity

## Abstract

High frequencies of loss of heterozygosity (LOH) in chromosome 11q22-qter have been observed in various malignancies, including breast cancer. Previous studies on breast carcinomas by Winqvist et al (*Cancer Res* **55**: 2660–2664) have indicated that a survival factor gene is located in band 11q23, and that the highly informative microsatellite polymorphism at the APOC3 locus would be a suitable tool to perform more extensive LOH studies. In this European multicentre study, we have examined the occurrence of APOC3 LOH and evaluated the effect of LOH of this chromosomal subregion on the clinical behaviour of the disease in a cohort of 766 breast cancer patients in more detail. LOH for APOC3 was found in 42% of the studied tumours, but it was not found to be significantly associated with any of the studied clinical variables, including cancer-specific survival time or survival time after recurrent/metastatic disease. According to the present findings, the putative survival factor gene on 11q23 is not located close enough to the APOC3 gene, but apparently at a more proximal location. © 1999 Cancer Research Campaign


*British Journal of Cancer* (1999) **80**(5/6), 879–882
© 1999 Cancer Research Campaign
Article no. bjoc.1998.0435

### European multicenter study on LOH of APOC3 at 11q23 in 766 breast cancer patients: relation to clinical variables

**V Launonen[1], K Laake[4], P Huusko[1], D Niederacher[5], MW Beckmann[5], RB Barkardottir[6], EK Geirsdottir[6], J Gudmundsson[6], P Rio[7], Y-J Bignon[8], S Seitz[9], S Scherneck[9], I Bièche[10], M-H Champème[10], D Birnbaum[11], G White[12], J Varley[12], M Sztán[13], E Olah[13], A Osorio[14], J Benitez[14], N Spurr[15]*, N Velikonja[16], B Peterlin[16], Å Borg[17], A-M Cleton-Jansen[18], P Devilee[18], R Bloigu[2], R Lidereau[10], A-L Børresen-Dale[4], R Winqvist[1,3], and the Breast Cancer Somatic Genetics Consortium**

Departments of [1]Clinical Genetics, [2]Mathematical Science/Statistics and [3]Oncology and Radiotherapy, University of Oulu/Oulu University Hospital, Oulu, Finland; [4]Department of Genetics, Institute for Cancer Research, The Norwegian Radium Hospital, Oslo, Norway; [5]Department of Obstetrics and Gynecology, Heinrich-Heine-University, Düsseldorf, Germany; [6]Laboratory of Cell Biology, Department of Pathology, University Hospital of Iceland, Reykjavik, Iceland; [7]INSERM U 484 Clermont-Ferrand, France; [8]Laboratoire d'Oncologie Moléculaire INSERM CRI 9502 and EA 2145, Centre Jean Perrin, Clermont-Ferrand, France; [9]Department of Tumour Genetics, Max-Delbrück-Centrum, Berlin, Germany; [10]Centre René Huguenin, Saint-Clod, France; [11]Institut Paoli-Calmettes and U.119 INSERM, Marseille, France; [12]Section of Molecular Genetics, Paterson Institute for Cancer Research, Manchester, UK; [13]National Institute of Oncology, Budapest, Hungary; [14]Department of Genetics, Fundación Jiménez Díaz, Madrid, Spain; [15]Human Genetic Resources, ICRF, Clare Hall, Laboratories, London, UK; [16]Division of Medical Genetics, Department of Obstetrics and Gynaecology, Ljubjana, Slovenia; [17]Institute of Oncology, University Hospital, Lund, Sweden; [18]Department of Pathology, Leiden University, Leiden, The Netherlands

**Summary** High frequencies of loss of heterozygosity (LOH) in chromosome 11q22-qter have been observed in various malignancies, including breast cancer. Previous studies on breast carcinomas by Winqvist et al (*Cancer Res* **55**: 2660–2664) have indicated that a survival factor gene is located in band 11q23, and that the highly informative microsatellite polymorphism at the APOC3 locus would be a suitable tool to perform more extensive LOH studies. In this European multicentre study, we have examined the occurrence of APOC3 LOH and evaluated the effect of LOH of this chromosomal subregion on the clinical behaviour of the disease in a cohort of 766 breast cancer patients in more detail. LOH for APOC3 was found in 42% of the studied tumours, but it was not found to be significantly associated with any of the studied clinical variables, including cancer-specific survival time or survival time after recurrent/metastatic disease. According to the present findings, the putative survival factor gene on 11q23 is not located close enough to the APOC3 gene, but apparently at a more proximal location.

**Keywords:** breast cancer; chromosome 11q23; loss of heterozygosity

Loss of heterozygosity (LOH) for a specific chromosome region may indicate the presence of a tumour suppressor gene (TSG). Studies on tumour LOH have therefore been helpful to identify many TSGs (Bièche and Lidereau, 1995). High incidences of LOH of the 11q22-qter chromosome region have been seen in breast cancer (Hampton et al, 1994; Gudmundsson et al, 1995; Negrini et al, 1995; Winqvist et al, 1995; Kerangueven et al, 1997; Laake et al, 1997) and also in several other human malignancies (Herbst et al, 1995; Rasio et al, 1995; Gabra et al, 1996; Hui et al, 1996). In addition, it has been shown that chromosome 11 can suppress tumorigenicity when transferred to breast cancer (Negrini et al, 1994; Phillips et al, 1996) and melanoma cell lines (Robertson et al, 1996).

The distal half of chromosome 11q contains several genes indicated to be involved in tumorigenesis; e.g. ATM (the ataxia telangiectasia disorder gene at 11q23.1), DDX10 (a putative RNA helicase gene at 11q23.1), MLL1 (a gene at 11q23 frequently rearranged in acute leukaemia), LOH11CR2A (a potential tumour suppressor gene at 11q23) and CHEK1 (a gene at 11q24 encoding a protein kinase required for the DNA damage checkpoint function) (Ziemin-van der Poel et al, 1991; Savitsky et al, 1995, 1996; Furnari et al, 1997; Monaco et al, 1997; Sanchez et al, 1997).

LOH at 11q23 has been reported in association with poor post-metastatic survival in breast cancer (Winqvist et al, 1995). The crucial region of LOH seemed to map between loci D11S35 (11q22) and APOC3 (apolipoprotein C-3 at 11q23) (Hampton et al, 1994), in a chromosomal segment of less than 17 Mb (Arai et al, 1996). In the initial study of a Finnish breast cancer cohort, APOC3 appeared to be the most suitable marker for more careful examinations of the clinical effects of LOH of the 11q23 chromosomal region. Therefore, in the present European multicentrestudy, we analysed tumour and normal tissue pairs of 766 primary breast cancer patients from 11 countries to investigate the association between LOH at the APOC3 locus and clinical variables in greater detail.

### MATERIALS AND METHODS

Altogether 766 primary tumour and normal tissue pairs from breast cancer patients were collected for the LOH analysis. The

*Received 6 August 1998*
*Revised 4 December 1998*
*Accepted 4 December 1998*

*Correspondence to:* R Winqvist

*Present address: SmithKline Beecham Pharmaceuticals Research and Development, Harlow, Essex, UK

**Table 1** Clinical characteristics of the studied European breast cancer cohort

| Country | Number of patients | Period of collection | Mean age of disease onset (range) | Mean follow-up time in months (range) | Positive family history (cases) | Bilateral disease (cases) | Recurrent/metastatic disease course (cases) |
|---|---|---|---|---|---|---|---|
| Finland | 85 | 1988–90 | 57 (29–84) | 75 (5–117) | 7 | 5 | 39 |
| France I | 46 | 1993–95 | 60 (35–87) | – | – | 2 | – |
| France II | 48 | 1978–87 | 53 (27–86) | 123 (11–198) | – | 0 | 25 |
| France III | 61 | 1988–96 | 61 (31–84) | 32 (3–104) | 31 | 5 | 5 |
| Germany I | 52 | 1993–95 | 58 (33–82) | 17 (2–31) | – | – | 2 |
| Germany II | 76 | 1992–96 | 55 (29–83) | 26 (6–166) | 23 | 8 | 13 |
| Hungary | 41 | 1980–94 | 52 (33–76) | 42 (4–160) | – | 5 | 18 |
| Iceland | 70 | 1985–93 | 58 (29–95) | 49 (2–92) | – | – | 27 |
| The Netherlands[a] | 11 | 1986–92 | 59 (39–73) | 52 (7–103) | – | – | 11 |
| Norway | 160 | 1984–94 | 60 (28–87) | 55 (1–122) | 23 | 8 | 48 |
| Slovenia | 17 | 1993–96 | 50 (37–68) | 33 (24–48) | 3 | 0 | 1 |
| Spain | 21 | 1994–96 | 55 (34–83) | 11 (1–43) | 4 | 1 | 3 |
| Sweden[a] | 15 | 1987–92 | 51 (35–73) | 55 (7–117) | 1 | 2 | 15 |
| UK I | 19 | 1989–90 | 57 (36–79) | 54 (21–70) | – | 2 | 6 |
| UK II | 44 | 1987–88 | 58 (30–85) | 69 (8–123) | 5 | 4 | 18 |
| All cases | 766 | 1978–96 | 57 (27–95) | 57 (1–198) | 97 | 42 | 231 |

– = information not available. [a] = selected cohort (all patients displayed cancer metastasis during the indicated follow-up time).

**Table 2** Summary of clinical variables and LOH of APOC3 at 11q23 in the studied European breast cancer cohort of 766 cases at time of diagnosis

| Variable | No. of cases | No. of informative cases (%) | APOC3 LOH % |
|---|---|---|---|
| Tumour size | | 703 | |
| < 2 cm | 271 (39) | | 39 |
| 2–5 cm | 353 (50) | | 41 |
| > 5 cm | 79 (11) | | 53 |
| Positive node status | | 675 | |
| Yes | 277 (41) | | 42 |
| No | 398 (59) | | 41 |
| Distant metastasis | | 592 | |
| Yes | 28 (5) | | 56 |
| No | 564 (95) | | 41 |
| Oestrogen receptor status | | 548 | |
| Positive | 355 (65) | | 45 |
| Negative | 193 (35) | | 38 |
| Progesterone receptor status | | 541 | |
| Positive | 315 (58) | | 44 |
| Negative | 226 (42) | | 42 |
| Grade | | 397 | |
| I | 52 (13) | | 43 |
| II | 196 (49) | | 39 |
| III | 149 (38) | | 51 |
| Histology | | 727 | |
| Ductal | 564 (78) | | 44 |
| Lobular | 111 (15) | | 39 |
| Medullar | 16 (2) | | 50 |
| Tubular | 16 (2) | | 38 |
| Mucinous | 5 (1) | | 60 |
| Other | 15 (2) | | 31 |

studied patient material was collected from 15 different research centres, representing 11 European countries (Table 1). Clinical characteristics are summarized in Tables 1 and 2. The patients studied were diagnosed with primary breast cancer between 1978 and 1996. The mean age of disease onset was 57 years (range 27–95). The mean follow-up time of those patients still alive was 57 months (range 1–198). Information about the family history of breast/ovarian cancer (in two or three first-degree relatives) and disease bilaterality was also collected. Tumours were classified according to size (< 2 cm, 2–5 cm or > 5 cm in diameter), histology, histoprognostic grade, and oestrogen receptor and progesterone receptor status. In addition, information about node and metastasis status at the time of diagnosis and possible adjuvant cancer therapy was obtained. Twenty-eight (4.7%) of the patients presented with metastatic disease at the time of diagnosis, while an additional 203 patients displayed local and/or distal tumour recurrence/metastasis during the clinical follow-up time. All cases from Sweden and the Netherlands were selected according to a metastatic disease course during the clinical follow-up time.

DNA from tumour tissue (fresh or paraffin-embedded) and corresponding normal tissue (blood, fresh or paraffin-embedded tissue) was extracted using standard phenol–chloroform protocols. Only a minority of the tissue material used was from paraffin-embedded tissues (2%, (17/766), all cases from Slovenia). In these cases DNA was extracted using the methods described by Sarkar (1995). The LOH analysis using the highly informative mononucleotide repeat microsatellite marker APOC3 (Bhattacharya et al, 1991) was performed either in Oulu or in the research centre from where the tissue material was collected. Polymerase chain reaction (PCR) was performed mainly as described previously (Bhattacharya et al, 1991), with some slight modifications, e.g. alternatively using radioactive or non-radioactive PCR methods. In the radioactive protocols we used either direct incorporation of [$\alpha$-$^{32}$]-dCTP or [$^{32}$P]-end-labelled PCR primers. Two different non-radioactive PCR protocols were used. One utilized silver staining to visualize the PCR products after electrophoresis. The other method used fluorescent-labelled PCR primers and an automated DNA sequencer for fragment analysis (Pharmacia, ALF; Perkin-Elmer Applied Biosystems, model 373A). In all cases, the PCR products were resolved by electrophoresis on 6–7% denaturing polyacrylamide gels. The evaluation of LOH status was performed in the radioactive method by comparing the normal and tumour tissue allele intensity ratios of the autoradiograms. Each case was evaluated by at least two independent viewers who

 *© Cancer Research Campaign 1999*

**Table 3** LOH at APOC3 (11q23) in different European populations

| Country | APOC3 LOH % (cases with LOH/ informative cases) |
|---|---|
| Finland | 41 (31/75) |
| France I | 56 (20/36) |
| France II | 41 (14/34) |
| France III | 38 (12/32) |
| Germany I | 37 (17/46) |
| Germany II | 32 (18/57) |
| Hungay | 52 (14/27) |
| Iceland | 33 (20/60) |
| The Netherlands[a] | 89 (8/9) |
| Norway | 45 (56/125) |
| Slovenia | 29 (4/14) |
| Spain | 58 (7/12) |
| Sweden[a] | 64 (9/14) |
| UK I | 44 (8/18) |
| UK II | 37 (14/38) |
| All cases | 42 (252/597) |
| Sporadic cases | 43 (122/282) |
| Familial cases | 31 (22/70) |
| No information | 44 (108/245) |

[a]Selected cohort.

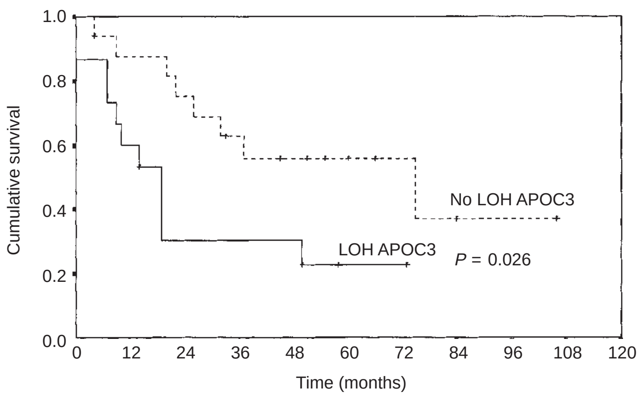

**Figure 1** Kaplan–Meier estimates for survival curves of breast cancer patients after diagnosis of recurrence/metastasis in the Finnish cohort. The patients with LOH at APOC3 in their tumours showed slightly reduced survival times ($P = 0.026$, log-rank test)

compared their observations. In the fluorescent-labelled microsatellite analysis the data were analysed with appropriate software (Pharmacia, Fragment Manager FM1.1; Perkin Elmer Applied Biosystems, Gene Scan) by comparing normal and tumour tissue allele peak sizes, heights and area ratios. In both methods, intensity or signal ratio differences of at least 25% (depending on the proportion of tumour cells or method used) were considered sufficient for LOH assignment.

The Fisher's two-tailed exact test, the Mann–Whitney test, the Pearson test and the stepwise logistic regression analysis methods were used for the statistical evaluation of associations between LOH at 11q23 and clinical findings. Survival curves calculated by Kaplan–Meier estimations were compared according to the log-rank test. $P$-values below 0.01 were considered significant. Only subpopulations with more than 40 breast cancer cases were analysed independently.

### RESULTS AND DISCUSSION

The results obtained from LOH analysis of the 11q23 subregion at APOC3 are summarized in Tables 2 and 3. The APOC3 marker was informative for determining LOH status in 78% of the studied cases. LOH was observed in 42% of the tumours. The LOH frequencies of the larger unselected subpopulations (with at least 40 patients) varied from 32% to 56%. In addition to the studied cohort, differencies seen in the LOH incidences could also be due to the analysis methods used. The LOH frequency was 47% in the patients with recurrent/metastatic disease (data not shown). Interestingly, primary tumours of the two small populations from Sweden and The Netherlands that had been selected for the presence of a metastatic disease course displayed the highest LOH frequencies (64% and 89% respectively).

According to the statistical analysis, no strong association was found between primary tumour LOH at APOC3 and any of the studied clinical variables (tumour size, node or metastasis status, histoprognostic grade, histology type, oestrogen or progesterone status, adjuvant therapy, bilaterality vs unilaterality, and family history of cancer). Correlation was seen between occurrence of LOH and disease onset at a higher age ($P = 0.014$). This could reflect the observation that the frequency of LOH was 43% (122/282) for the sporadic cases, but only 31% (22/70) for the cases with a positive family history of cancer, known usually to have earlier onset of the disease. No association was seen between LOH and cancer-specific survival time ($P = 0.146$) or survival after diagnosis of recurrence/metastasis ($P = 0.987$).

Although most of the studied cohorts represent cases from a certain time period, they seemed to be clinically heterogeneous. Nevertheless, we analysed separately all subpopulations consisting of at least 40 patients. Only a slight correlation was observed between LOH at APOC3 and a positive oestrogen status in the Germany II cohort ($P = 0.029$), a greater tumour size and a more advanced tumour grade in the France II cohort ($P = 0.026$ and $P= 0.024$ respectively), and a more advanced tumour grade in the sporadic cases of the Norwegian cohort ($P = 0.025$). We could not detect a significant association between LOH for APOC3 and cancer-specific survival times or survival after recurrence/metastasis in any of the studied subpopulations. Only in the Finnish cohort was a slight correlation between LOH and reduced survival times after disease recurrence/metastasis seen ($P = 0.026$) (Figure 1). However, the clinical association was found to be much weaker than in the initial analysis of the same cohort ($P = 0.0004$; Winqvist et al, 1995). The difference could be explained by the fact that in the current investigation, due to the longer follow-up time, a greater number of patients showed relapse. In the previous analysis, the follow-up time was approximately 3–5 years and, altogether, 28 patients displayed a metastatic disease. In this study, five more cases showed a metastatic disease course during the updated follow-up time (mean > 6 years, range 5–117 months). Interestingly, due to the prolonged follow-up time, the correlation between LOH at 11q23 and shortened post-metastatic survival time was not as strong as had been seen previously, suggesting that in the Finnish cohort the adverse effect on survival is mainly limited to the first 2–3 years after relapse.

The results presented here make it unlikely that the putative survival factor gene on 11q23 would be situated very close to the subregion containing the APOC3 gene. As our previous studies have indicated that the crucial region of LOH seems to be located between the D11S35 and APOC3 loci (Hampton et al, 1994;

© Cancer Research Campaign 1999 

Winqvist et al, 1995), it is possible that the survival factor gene could be at a more proximal location. Interestingly, one additional subregion exhibiting LOH on chromosome 11q23 has been mapped closer to the DDX10 and ATM genes (Laake et al, 1997; Hui et al, 1996). The involovement of this subregion was confirmed by our parallel study on LOH of 11q23. Possible additional targets of LOH on chromosome 11q could include the CHEK1 and LOH11CR2A genes (Furnari et al, 1997; Sanchez et al, 1997; Monaco et al, 1997). However, LOH11CR2A gene mutations have not so far been detected in breast, ovarian or lung tumours (Monaco et al, 1997). As chromosome 11q seems to harbour multiple genes important for tumour development, it is likely that the size and number of deleted chromosomal segments could be important for determining their clinical effects.

In conclusion, LOH of 11q23 at APOC3 is a frequent finding in primary breast tumours, and it is even more common in the tumours of patients developing a more advanced disease. However, LOH was not seen to be strongly associated with any of the studied clinical variables, suggesting that the putative survival factor gene on 11q23 is not located in the immediate vicinity of the APOC3 locus, but more likely further proximal towards the chromosomal subregion harbouring the DDX10 and ATM genes.

### ACKNOWLEDGEMENTS

We gratefully acknowledge the many clinicians and pathologists who assisted in collecting the biological specimens and provided the necessary clinical information for this study. We also wish to thank the numerous breast cancer patients for making this study possible. The help of many skilful research technicians is also highly appreciated.

### REFERENCES

Arai Y, Hosoda F, Nakayama K and Ohki M (1996) A yeast artificial chromosome contig and NotI restriction map that spans the tumor suppressor gene(s) locus, 11q22.2–q23.3. *Genomics* **35**: 196–206

Bhattacharya S, Wilson TME, Wojciechowski AP, Volpe CP and Scott J (1991) Hypervariable polymorphism in the APOC3 gene. *Nucleic Acids Res* **17**: 4799

Bièche I and Lidereau R (1995) Genetic alterations in breast cancer. *Genes Chromosomes Cancer* **14**: 227–251

Furnari B, Rhind N and Russell P (1997) Cdc25 mitotic inducer targeted by Chk1 DNA damage checkpoint kinase. *Science* **277**: 1495–1497

Gabra H, Watson JEV, Taylor KJ, Mackay J, Leonard RCF, Steel CM, Porteous DJ and Smyth JF (1996) Definition and refinement of a region of loss of heterozygosity at 11q23.3–q24.3 in epithelial ovarian cancer associated with poor prognosis. *Cancer Res* **56**: 950–954

Gudmundsson J, Barkardottir RB, Eiriksdottir G, Baldursson T, Arason A, Egilsson V and Ingvarsson S (1995) Loss of heterozygosity at chromosome 11 in breast cancer: association of prognostic factors with genetic alterations. *Br J Cancer* **72**: 696–701

Hampton GM, Mannermaa A, Winqvist R, Alavaikko M, Blanco G, Taskinen PJ, Kiviniemi H, Newsham I, Cavenee WK and Evans GA (1994) Loss of heterozygosity in sporadic human breast carcinoma: a common region between 11q22 and 11q23.3. *Cancer Res* **54**: 4586–4589

Herbst RA, Larson A, Weiss J, Cavenee WK, Hampton GM and Arden KC (1995) A defined region of loss of heterozygosity a 11q23 cutaneus malignant melanoma. *Cancer Res* **55**: 2494–2496

Hui ABY, Lo K-W, Leung S-F, Choi PHK, Fong Y, Lee JCK and Huang DP (1996) Loss of heterozygosity on the long arm of chromosome 11 in nasopharyngeal carcinoma. *Cancer Res* **56**: 3225–3229

Kerangueven F, Eisinger F, Noguchi T, Allione F, Wargniez V, Eng C, Padberg G, Theillet C, Jacquemier J, Longy M, Sobol H and Birmbaum D (1997) Loss of heterozygosity in human breast carcinomas in the ataxia telangiectasia, Cowden disease and BRCAI gene regions. *Oncogene* **14**: 339–347

Laake K, Ødegård Å, Andersen TI, Bukholm IK, Kåresen R, Nesland JM, Ottestad L, Shiloh Y and Børresen-Dale A-L (1997) Loss of heterozygosity at 11q23.1 in breast carcinomas: indication for involvement of a gene distal and close to ATM. *Genes Chromosom Cancer* **18**: 175–180

Lindblom A, Sandelin K, Iselius L, Dumanski J, White I, Nordenskjöld M and Larsson C (1994) Predisposition for breast cancer in carriers of constitutional translocation 11q;22q. *Am J Hum Genet* **54**: 871–876

Monaco, C, Negrini M, Sozzi G, Veronese ML, Vorechovsky I, Godwin AK and Croce CM (1997) Molecular cloning and characterization of LOH11CR2A, a new gene within a refined minimal region of LOH at 11q23. *Genomics* **46**: 217–222

Negrini M, Sabbioni S, Possati L, Rattan S, Corallini A, Barbanti–Brodano G and Croce CM (1994) Suppression of tumorigenicity of breast cancer cells by microcell-mediated chromosome transfer: studies on chromosomes 6 and 11. *Cancer Res* **54**: 1331–1336

Negrini M, Rasio D, Hampton GM, Sabbioni S, Rattan S, Carter SL, Rosenberg AL, Schwartz GF, Shiloh Y, Cavenee WK and Croce CM (1995) Definition and refinement of chromosome 11 regions of loss of heterozygosity in breast cancer: identification of a new region at 11q23.3. *Cancer Res* **55**: 3003–3007

Phillips KK, Welch DR, Miele ME, Lee J-H, Wei LL and Weissman BE (1996) Suppression of MDA- MB-435 breast carcinoma cell metastasis following the introduction of human chromosome 11. *Cancer* **56**: 1222–1227

Rasio D, Negrini M, Manenti G, Dragani TA and Croce CM (1995) Loss of heterozygosity at chromosome 11q in lung adenocarcinoma: identification of three independent regions. *Cancer Res* **55**: 3988–3991

Robertson G, Coleman A and Lugo TG (1996) A malignant melanoma tumor suppressor on human chromosome 11. *Cancer Res* **56**: 4487–4492

Sanchez Y, Wong C, Thoma RS, Richman R, Wu Z, Piwnica-Worms H and Elledge SJ (1997) Conservation of the Chk1 checkpoint pathway in mammals: linkage of DNA damage to Cdk regulation through Cdc25. *Science* **277**: 1497–1501

Sarkar G (1995) *PCR in Neuroscience*, pp. 168–169. Academic Press: New York

Savitsky K, Bar-Shira A, Gilad S, Rotman G, Ziv Y, Vanagaite L, Tagle DA, Smith S, Uziel T, Stez S, Ashkenazi M, Pecker I, Frydman M, Harnik R, Patanjali SR, Simmons A, Clines GA, Sartiel A, Gatti RA, Chessa L, Sanal O, Lavin MF, Jaspers NGJ, Taylor AMR, Arlett CF, Miki T, Weissman SM, Lovett M, Collins FS and Shiloh Y (1995) A single ataxia telangiectasia gene with a product similar to PI-3 kinase. *Science* **268**: 1749–1753

Savitsky K, Ziv Y, Bar–Shira A, Gilad S, Tagle DA, Smith S, Uziel T, Sfez S, Nahmias J, Sartiel A, Eddy RL, Shows TB, Collins FS, Shiloh Y and Rotman G (1996) A human gene (DDX10) encoding a putative DEAD-box RNA helicase at 11q22-q23. *Genomics* **33**: 199–206

Winqvist R, Hampton GM, Mannermaa A, Blanco G, Alavaikko M, Kiviniemi H, Taskinen PJ, Evans GA, Wright FA, Newsham I and Cavenee WK (1995) Loss of heterozygosity for chromosome 11 in primary human breast tumors is associated with poor survival after metastasis. *Cancer Res* **55**: 2660–2664

Ziemin-van der Poel S, McCable NR, Gill HJ, Espinosa III R, Patel Y, Harden A, Rubinelli P, Smith SD, LeBeau MM, Rowley JD and Diaz MO (1991) Identification of a gene, MLL, that spans the breakpoint in 11q23 translocations associated with human leukemias. *Proc Natl Acad Sci USA* **88**: 10735–10739

 *© Cancer Research Campaign 1999*
